# Preemptive mechanical circulatory support in ventricular tachycardia ablation: A systematic review and meta-analysis

**DOI:** 10.1007/s10840-026-02445-0

**Published:** 2026-09-22

**Authors:** David T. Zhang, Mriganka Nerkar, Saborny Mahmud, Abhijeet Singh, Ibrahim O. Almasry, Roger Fan, Eric J. Rashba

**Affiliations:** 1 Electrophysiology Section, Division of Cardiology, Department of Medicine, Weill Cornell Medicine, New York, NY USA; 2 Renaissance School of Medicine, Stony Brook University, Stony Brook, NY USA; 3 Electrophysiology Section, Division of Cardiology, Department of Medicine, Stony Brook Medicine, Stony Brook, NY USA

**Keywords:** Mechanical circulatory support, Ventricular tachycardia, Catheter ablation

## Abstract

**Background:**

The benefit of preemptive mechanical circulatory support (MCS) for ventricular tachycardia (VT) ablation remains unclear. This meta-analysis compared the efficacy and safety of preemptive MCS to standard care for VT ablation.

**Methods:**

An unrestricted literature search of PubMed, Embase, and Web of Science identified studies on preemptive MCS for VT ablation. MCS included percutaneous left ventricular assist devices or extracorporeal membrane oxygenation. The primary endpoint was all-cause mortality; secondary endpoints included VT recurrence, procedural time, procedural complications (vascular access, bleeding, stroke, pericardial effusion, heart failure, myocardial infarction), and 30-day major adverse cardiovascular events (MACE).

**Results:**

Eight studies with 2,262 patients (414 with MCS, 1,848 without) were included. Mean follow-up was 15.8 months, mean age was 62.6 years, 12.5% were women, and mean PAINESD scores were 15.7 in the MCS group and 10.5 in the no-MCS group. There was no significant difference in all-cause mortality (OR 1.58, 95% CI 0.60–4.19, *p* = 0.36, I²=81%) or VT recurrence (OR 1.03, 95% CI 0.68–1.56, *p* = 0.90, I²=46%). Preemptive MCS was associated with longer procedural times (mean difference 46.0 min, 95% CI 11.1–80.8, *p* = 0.01, I²=86%), more procedural complications—driven by vascular access and pericardial effusion (OR 2.25, 95% CI 1.26–4.03, *p* = 0.006, I²=33%). Thirty-day MACE was not uniformly defined across studies and was therefore not pooled.

**Conclusions:**

Preemptive MCS was not associated with a significant difference in all-cause mortality or VT recurrence in patients undergoing VT ablation but was associated with longer procedures, and more procedural complications. The certainty of evidence was very low, reflecting the observational study design, baseline imbalance between groups, and heterogeneity; these findings do not preclude the selective use of MCS in high-risk patients and require confirmation in randomized trials.

**Graphical Abstract:**

**Figure:**
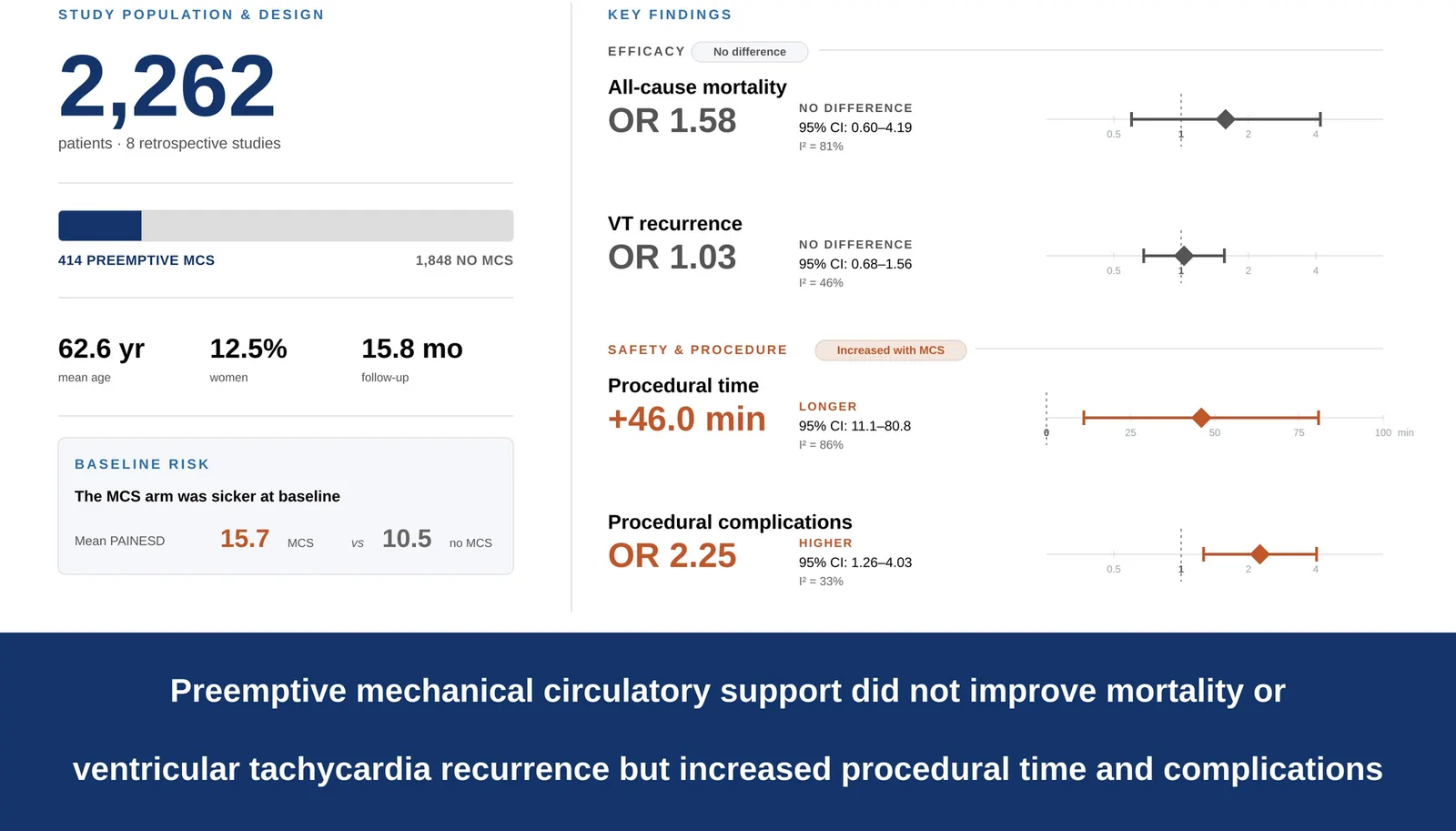
Across 8 studies of 2,262 patients, preemptive mechanical circulatory support in ventricular tachycardia (VT) ablation was not associated with lower mortality or VT recurrence but was associated with longer procedures and more complications

**Supplementary Information:**

The online version contains supplementary material available at https://doi.org/10.1007/s10840-026-02445-0.

## Introduction

Radiofrequency catheter ablation is an established strategy to treat ventricular tachycardia (VT) refractory to antiarrhythmic medications [1]. VT ablation has been shown to reduce arrhythmia recurrence, ICD shocks, and arrhythmia-related hospitalizations [2]. Patients undergoing ablation for scar-related VT often have several comorbidities and procedural factors that can lead to periprocedural hemodynamic instability, such as volume overload and hypotension related to underlying heart failure and its medical therapy [2, 3]. Furthermore, a major procedural challenge is that sustained VT is often not hemodynamically tolerated, limiting the ability to perform activation and entrainment mapping to accurately localize the arrhythmogenic substrate [4]. Consequently, acute periprocedural hemodynamic decompensation may require advanced hemodynamic support or premature termination of the ablation procedure [5]. Acute hemodynamic decompensation during VT ablation has also been associated with increased procedural complications and worse clinical outcomes [6].

To mitigate this risk, preemptive mechanical circulatory support (MCS) has been proposed using devices such as intra-aortic balloon pump (IABP), percutaneous left ventricular assist device (pLVAD), and venoarterial extracorporeal membrane oxygenation (VA-ECMO) to provide hemodynamic stabilization and facilitate sustained VT mapping and more complete substrate modification [7]. Prior literature has demonstrated mixed results on the risks and benefits of preemptive MCS in these settings. Some hemodynamic benefits include decreased myocardial oxygen consumption, ventricular wall stress, and cardiac filling pressures and volumes [7]. Risk stratification tools such as the PAINESD score have also been developed to identify patients at increased risk of acute hemodynamic collapse during VT ablation and may help guide the use of prophylactic MCS [6, 8]. However, other studies have suggested there may be increased procedural complications and interference between MCS and three-dimensional electroanatomic mapping systems [5]. As a result, there remains no clear consensus regarding the role of preemptive MCS in patients undergoing VT ablation. We conducted a systematic review and meta-analysis to evaluate the benefits and risks of preemptive MCS for hemodynamic stabilization in patients undergoing VT ablation.

## Methods

### Data search

The systematic review was conducted in May 2026 using a preplanned protocol in accordance with the Preferred Reporting Items for Systematic Reviews and Meta-Analyses (PRISMA) statement. A review protocol was not prospectively registered. Databases searched included PubMed, Web of Science, and Embase. Search terms included combinations of the following keywords and MeSH terms: (“Ablation”) AND (“mechanical circulatory support” OR “ecmo” OR “impella”) NOT (Review[publication type]). The search was not restricted by time or publication status.

In addition, reference lists of selected articles were screened for relevant citations that may have been missed by electronic searches. All study designs were eligible for inclusion, including retrospective studies, prospective cohorts, registries, and randomized controlled trials. The protocol was developed prior to data extraction but was not prospectively registered.

### Study selection

Studies were screened independently by two investigators (MN, SM) at the level of title and abstract, followed by full-text review for eligibility. Disagreements were resolved by consensus with involvement of a third investigator (DTZ).

Inclusion criteria were studies evaluating the use of preemptive MCS during ventricular tachycardia ablation and reporting clinical outcomes comparing patients who received MCS with those who underwent ablation without MCS. MCS included percutaneous left ventricular assist devices (pLVAD) or extracorporeal membrane oxygenation (ECMO) used prophylactically prior to or at the start of the ablation procedure. Intra-aortic balloon pump (IABP) was excluded as a source of MCS, as IABP provides relatively limited hemodynamic support compared with other percutaneous MCS devices [7]. 

Exclusion criteria included review articles, case reports, conference abstracts without sufficient outcome data, studies not reporting comparative outcomes, or studies evaluating only rescue MCS after hemodynamic collapse. In all included cohorts, mechanical circulatory support was in place before or at the start of the ablation procedure, including studies in which support had been established for cardiogenic shock or refractory electrical storm prior to ablation. Studies not published in English were also excluded. When multiple studies reported overlapping patient cohorts from the same center, we retained the study providing the most complete data for the prespecified outcomes to avoid duplicate inclusion of patients.

### Data extraction

Data extraction was performed independently by two investigators, and discrepancies were resolved by consensus. A standardized data collection form was used to record study characteristics including publication year, study design, number of participants, patient demographics, type of MCS, and follow-up duration.

Clinical outcomes extracted included all-cause mortality, VT recurrence, procedural time, procedural complications, and 30-day major adverse cardiovascular events (MACE). All-cause mortality was captured at the follow-up interval reported by each study, which varied across studies and did not correspond to a uniform time point (Supplementary Table 1). Procedural complications included vascular access complications, bleeding, stroke, pericardial effusion, heart failure, and myocardial infarction. For the pooled analysis, procedural complications were counted as the number of patients with at least one complication in each arm rather than the total number of complication events. Study quality was assessed independently by two investigators using the Newcastle–Ottawa Scale for cohort studies, which evaluates the selection, comparability, and outcome domains for a maximum of nine stars, with discrepancies resolved by consensus. The certainty of evidence for each outcome was appraised using the Grading of Recommendations Assessment, Development and Evaluation (GRADE) framework, with observational evidence beginning at low certainty and rated for risk of bias, inconsistency, indirectness, imprecision, and publication bias.

### Statistical analysis

Meta-analysis of summary statistics from individual studies was performed using Cochrane Review Manager Version 5.4. Dichotomous outcomes were expressed as odds ratios (OR) with 95% confidence intervals (CI), while continuous outcomes were expressed as mean differences (MD) with 95% CI.

A random-effects model was used to account for potential heterogeneity among included studies. Heterogeneity was assessed using the I² statistic, with values of 0–40% considered low heterogeneity, 40–60% moderate heterogeneity, 60–90% substantial heterogeneity, and > 90% considerable heterogeneity. Statistical significance was defined as a two-sided p-value < 0.05.

To explore sources of heterogeneity, we performed a subgroup analysis restricted to studies using percutaneous left ventricular assist devices; studies using venoarterial extracorporeal membrane oxygenation or mixed device strategies were too few to pool separately. We further performed leave-one-out sensitivity analyses, sequentially omitting each study, to evaluate the influence of individual studies on the pooled estimates and on between-study heterogeneity.

## Results

### Literature search and study selection

**Fig. 1:**
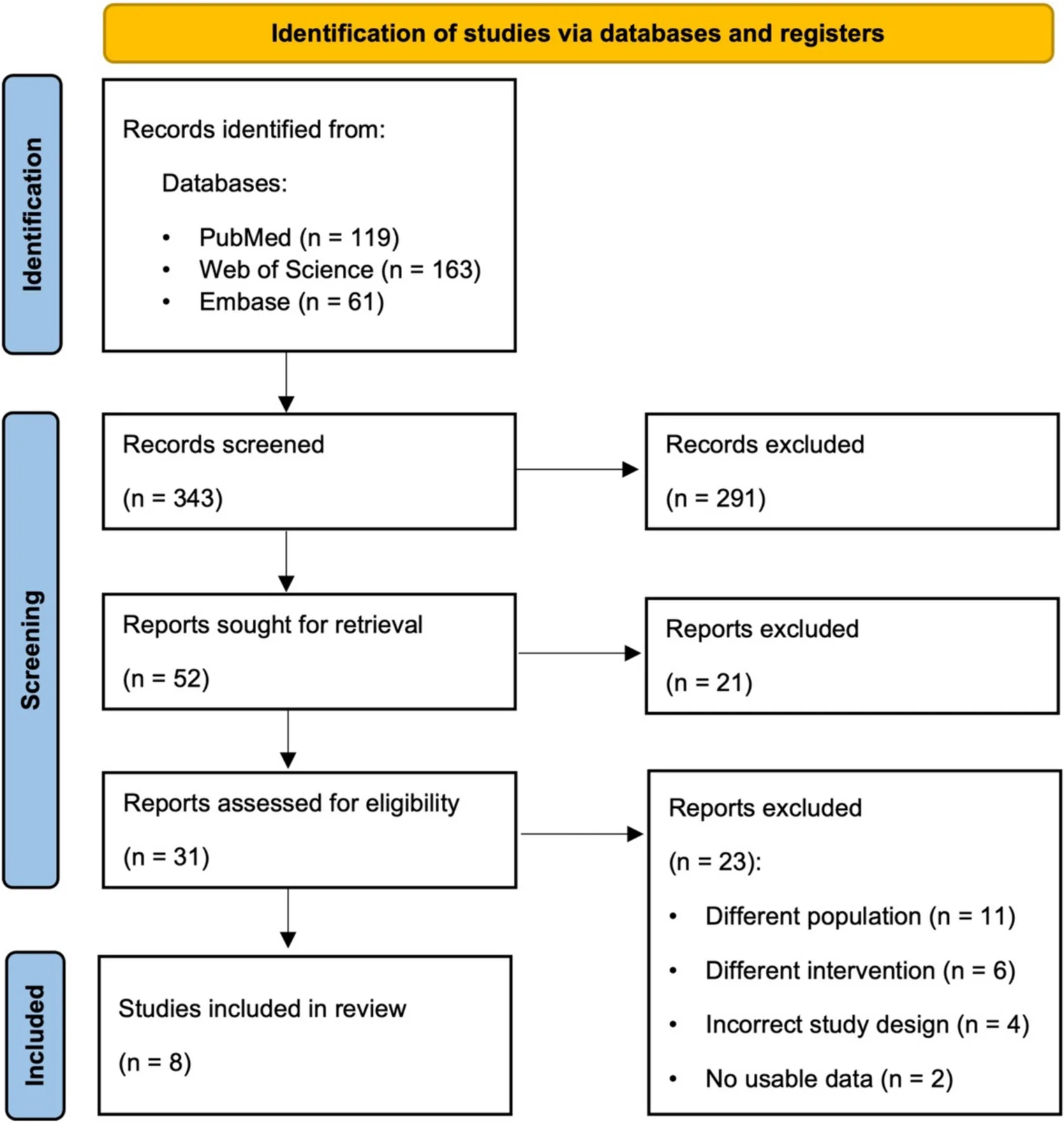
Flow diagram depicting study selection for meta-analysis inclusion according to PRISMA statement

The initial literature search identified 343 potentially relevant studies across the three databases. After removal of duplicate records and screening of titles and abstracts, 31 studies underwent full-text review for eligibility. Eight studies met the inclusion criteria and were included in the systematic review and meta-analysis, comprising a total of 2,262 patients undergoing VT ablation [9–16]. Of these, 414 patients received preemptive MCS, while 1,848 patients underwent ablation without MCS. The process of study identification and selection is summarized in the PRISMA flow diagram (Fig. 1). All eight studies were retrospective.

### Study, patient, and procedural characteristics

Baseline characteristics of the included studies are summarized in Table 1. The mean age of patients included in the analysis was 62.6 years, and 12.5% of patients were women. The mean follow-up duration across studies was 15.8 months. The MCS group carried a higher baseline predicted risk than the no-MCS group, with a mean PAINESD score of 15.7 compared with 10.5 and a lower mean left ventricular ejection fraction (Table 1). MCS strategies included percutaneous left ventricular assist devices and extracorporeal membrane oxygenation, which were used preemptively prior to or at the start of the VT ablation procedure.

**Table 1:** Baseline patient and procedural characteristics of the included studies

Study	MCS device	Follow-up, mo	Patients, *n*		Age, y		Women, *n* (%)		LVEF, %		PAINESD	
			MCS	No MCS	MCS	No MCS	MCS	No MCS	MCS	No MCS	MCS	No MCS
Aryana 2014 [9]	pLVAD	19	34	34	64 ± 12	67 ± 12	6 (18%)	2 (6%)	30 ± 11	34 ± 9	NR	NR
Bunch 2012 [10]	pLVAD	9	13	18	59.7	62.5	1 (8%)	3 (17%)	20	25	NR	NR
Grimaldi 2021 [11]	VA-ECMO	24	31	31	66 ± 10	69 ± 8	3 (10%)	1 (3%)	30 ± 10	30 ± 8	21 ± 7	22 ± 5
Kataoka 2023 [12]	mixed MCS	3	6	14	71	76	3 (50%)	1 (7%)	31 ± 8.4	39.8 ± 12	15.3 ± 4.4	10.1 ± 3.1
Kusa 2017 [13]	pLVAD	7	109	85	64 ± 11	61 ± 15	8 (7%)	17 (20%)	26 ± 10	39 ± 16	NR	NR
Muser 2018 [14]	pLVAD	9	75	75	65 ± 12	64 ± 14	2 (3%)	5 (7%)	27 ± 10	27 ± 12	13	12
Sroubek 2023 [15]	pLVAD	15.4	41	41	66.6 ± 9	66.7 ± 11.5	4 (10%)	3 (7%)	25	24	18	16
Turagam 2017 [16]	mixed MCS	17.3	105	1550	63.6 ± 11.2	61.7 ± 14	15 (14%)	208 (13%)	25.4 ± 11.9	35.6 ± 12.9	15.3 ± 6.2	10.1 ± 6.3
**Pooled / Total**	**—**	**15.8**	**414**	**1848**	**64.4**	**62.2**	**42 (10%)**	**240 (13%)**	**26**	**35**	**15.7**	**10.5**

Values are mean ± SD or n (%) unless otherwise indicated. Abbreviations: *LVEF* = left ventricular ejection fraction; *MCS* = mechanical circulatory support; *NR* = not reported; *PAINESD* = pulmonary disease, age, ischemic cardiomyopathy, New York Heart Association class, ejection fraction, electrical storm, diabetes; *pLVAD* = percutaneous left ventricular assist device; *VA-ECMO* = veno-arterial extracorporeal membrane oxygenation; *VT *= ventricular tachycardia

### Study quality

Study quality, assessed using the Newcastle–Ottawa Scale, was moderate to high, with scores ranging from five to nine (Table 2); the comparability domain accounted for most of the potential for bias, reflecting the absence of adjustment for baseline differences between groups in the majority of studies. The certainty of evidence, appraised using the GRADE framework, was very low for all outcomes (Table 3), reflecting the observational design of the included studies together with between-study heterogeneity and imprecision.

A

**Table 2:** Newcastle–Ottawa Scale assessment of study quality

Study	S1	S2	S3	S4	Comparability	O1	O2	O3	Total (/9)
Aryana 2014 [9]	★	★	★	★	★	★	★	★	8
Bunch 2012 [10]	★	★	★	★	★	★	★	–	7
Grimaldi 2021 [11]	★	★	★	★	★	★	★	★	8
Kataoka 2023 [12]	–	★	★	★	–	★	–	★	5
Kusa 2017 [13]	★	★	★	★	★	★	★	★	8
Muser 2018 [14]	★	★	★	★	★★	★	★	★	9
Sroubek 2023 [15]	–	★	★	★	★	★	★	★	7
Turagam 2017 [16]	★	★	★	★	★	★	★	★	8

Newcastle–Ottawa Scale for cohort studies. Selection: S1, representativeness of the exposed (MCS) cohort; S2, selection of the non-exposed cohort; S3, ascertainment of exposure; S4, demonstration that outcomes of interest were not present at the start of the study. Comparability: adjustment or matching for baseline confounders, including baseline cardiac risk (maximum two stars). Outcome: O1, assessment of outcome; O2, adequacy of follow-up duration; O3, completeness of follow-up. One star is awarded per item except comparability (maximum two), for a maximum of nine. Scores of 7–9 denote high, 4–6 moderate, and below 4 low methodological quality

**Table 3:** GRADE summary of the certainty of evidence for each outcome

Outcome	Participants (studies)	Effect (95% CI)	Risk of bias	Inconsistency	Imprecision	Certainty (GRADE)
All-cause mortality	2,262 (8)	OR 1.58 (0.60–4.19)	Serious	Serious	Serious	Very low ⊕◯◯◯
VT recurrence	2,262 (8)	OR 1.03 (0.68–1.56)	Serious	Not serious	Not serious	Very low ⊕◯◯◯
Procedural complications	2,262 (8)	OR 2.25 (1.26–4.03)	Serious	Not serious	Not serious	Very low ⊕◯◯◯
Procedural time, min	2,180 (7)	MD 46.0 (11.1–80.8)	Serious	Serious	Not serious	Very low ⊕◯◯◯
30-day MACE	2,262 (8)	Not pooled	Serious	Serious	Serious	Very low ⊕◯◯◯

*GRADE* = Grading of Recommendations Assessment, Development and Evaluation. Certainty: ⊕⊕⊕⊕ high, ⊕⊕⊕◯ moderate, ⊕⊕◯◯ low, ⊕◯◯◯ very low. All outcomes were derived from observational studies and therefore began at low certainty. Risk of bias was rated down for the retrospective design and confounding by indication. Inconsistency was rated down for substantial statistical heterogeneity (I² greater than 60%). Imprecision was rated down when the confidence interval was wide and included both appreciable benefit and harm. Indirectness and publication bias were not serious for any outcome. *CI* = confidence interval; *MACE* = major adverse cardiovascular events; *MD* = mean difference; *OR* = odds ratio; *VT *= ventricular tachycardia

### Association between preemptive MCS and mortality

**Fig. 2:**
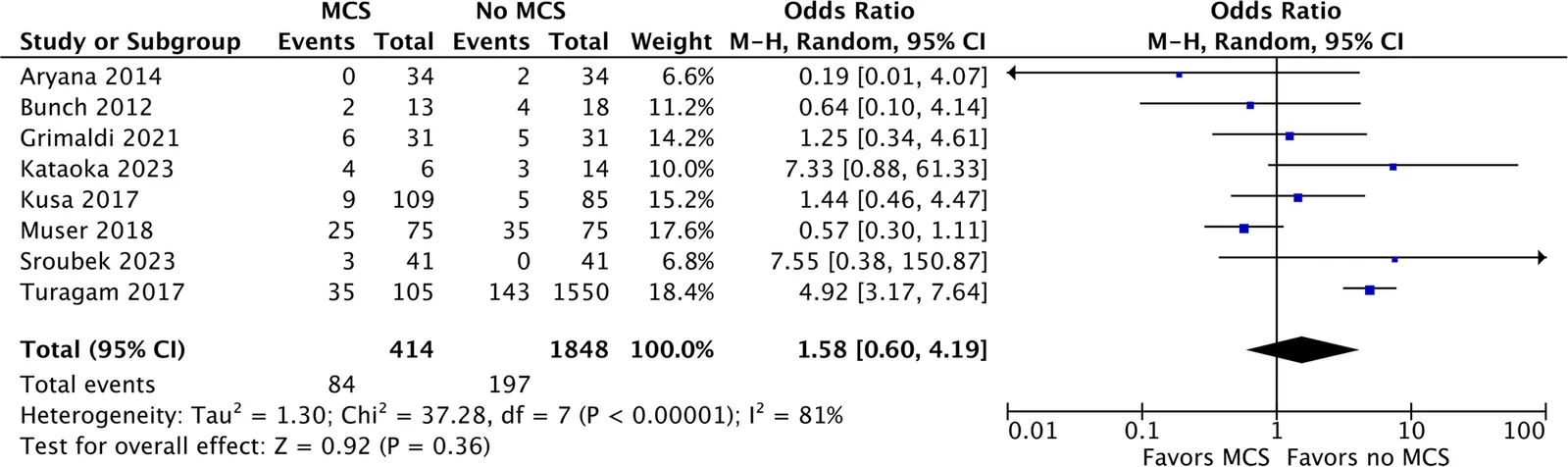
In patients undergoing VT ablation, forest plot of association of preemptive MCS on all-cause mortality

The association between preemptive MCS and all-cause mortality following VT ablation was evaluated across the eight included studies. The pooled analysis demonstrated no significant difference in all-cause mortality between patients receiving preemptive MCS and those undergoing ablation without MCS (OR 1.58; 95% CI 0.60–4.19; *p* = 0.36; I²=81%), reflecting substantial heterogeneity across studies (Fig. 2).

### VT recurrence

**Fig. 3:**
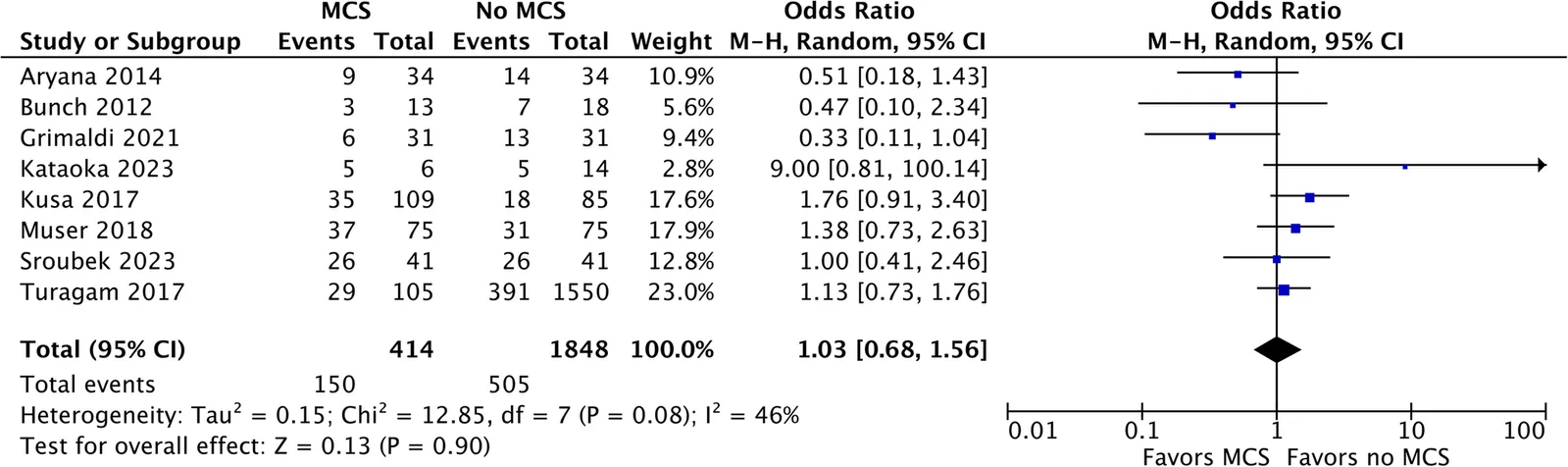
In patients undergoing VT ablation, forest plot of association of preemptive MCS on VT recurrence

Preemptive MCS was not associated with a significant difference in VT recurrence compared with ablation without MCS. The pooled odds ratio for VT recurrence was 1.03 (95% CI 0.68–1.56; *p* = 0.90; I²=46%), with moderate heterogeneity (Fig. 3).

### Procedural time

**Fig. 4:**
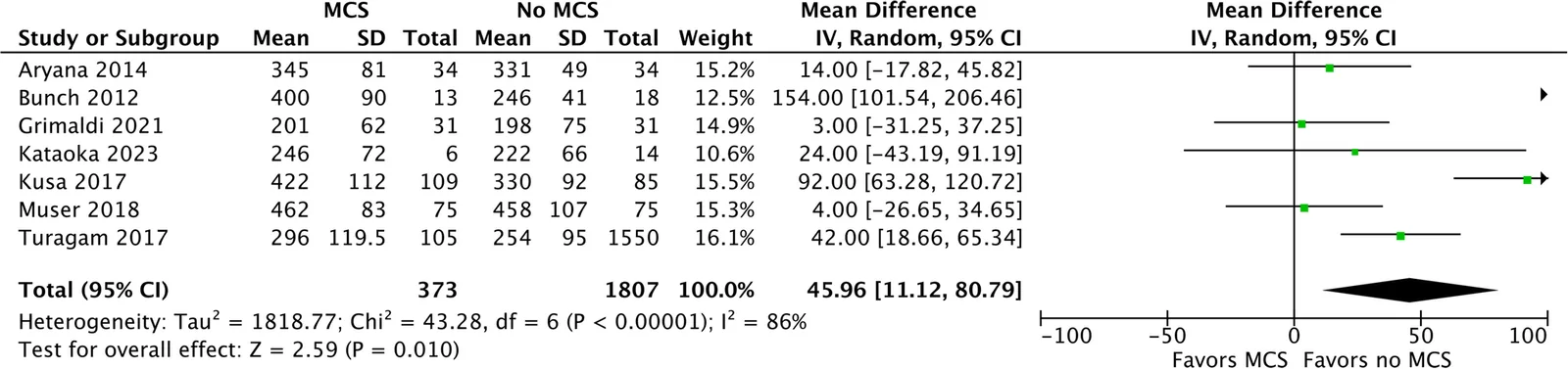
In patients undergoing VT ablation, forest plot of association of preemptive MCS on procedural time (minutes)

Preemptive MCS was associated with longer procedural duration compared with ablation without MCS. The pooled mean difference in procedural time was 46.0 min (95% CI 11.1–80.8; *p* = 0.01; I²=86%), with substantial heterogeneity across studies (Fig. 4).

### Procedural complications

**Fig. 5:**
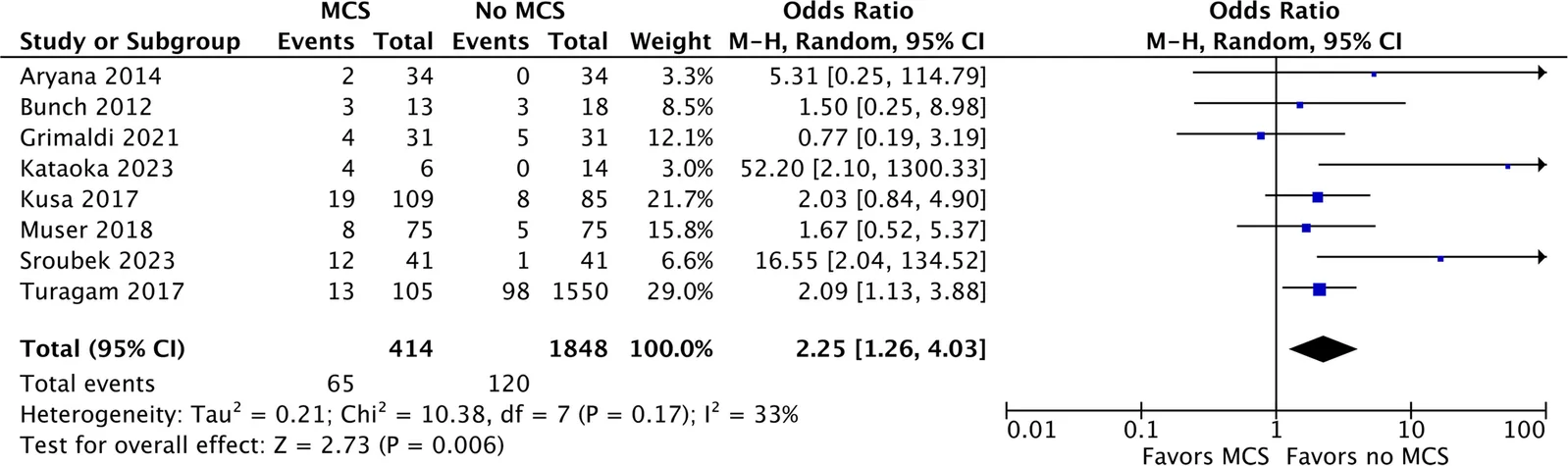
In patients undergoing VT ablation, forest plot of association of preemptive MCS on procedural complication rate

Patients receiving preemptive MCS experienced higher rates of procedural complications compared with those undergoing ablation without MCS. Complications were primarily driven by vascular access complications (18 versus 9 for MCS and no MCS, respectively) and pericardial effusion (18 versus 7). The pooled odds ratio for procedural complications was 2.25 (95% CI 1.26–4.03; *p* = 0.006; I²=33%), reflecting low heterogeneity (Fig. 5). The distribution of individual complication types is summarized in Table 4. The pooled odds ratio reflects the number of patients with at least one complication, whereas Table 4 tabulates individual complication events and may therefore exceed the number of affected patients, as a patient could experience more than one event. Vascular access complications were the most frequent event and were more common among patients receiving preemptive MCS (18 versus 9). When grouped by mechanism, vascular access- and device-related events, comprising access-site injury, bleeding, and device fracture, were more frequent with MCS (23 versus 9), concordant with the large-bore cannulation required for device placement. Systemic and other procedural complications, including heart failure or cardiogenic shock, were also more frequent with MCS (29 versus 13), a pattern that may in part reflect the higher baseline risk of patients selected for support.

**Table 4:** Breakdown of periprocedural complications by type

Complication	MCS (*n*)	No MCS (*n*)
Vascular and device-related		
Vascular access	18	9
Bleeding or hemolysis	4	0
Device fracture	1	0
**Subtotal**	**23**	**9**
**Systemic and other procedural**		
Pericardial effusion or tamponade	18	7
Heart failure or cardiogenic shock	4	3
Cardiac arrest	3	0
Venous thromboembolism	2	0
Stroke	2	1
Myocardial infarction	0	1
Nerve injury	0	1
**Subtotal**	**29**	**13**
**Total (itemized)**	**52**	**22**

Complications were grouped into vascular access-related and device-related events or systemic and other procedural events. One included study (Turagam et al.) reported only aggregate complication counts (13 with mechanical circulatory support and 98 without) without itemization and is therefore not represented in this breakdown

### Thirty-day major adverse cardiovascular events

Thirty-day MACE was not uniformly defined across the included studies, and the interval over which it was ascertained varied. Given this heterogeneity in outcome definition and the sensitivity of any pooled estimate to the exclusion of individual studies, MACE was not pooled and is summarized narratively. Across the studies reporting these events, MACE occurred numerically more often among patients receiving preemptive MCS than among those undergoing ablation without support, although this pattern was not consistent across studies. The certainty of this evidence was very low (Table 3).

### Subgroup and sensitivity analyses

In a subgroup analysis restricted to the five studies using percutaneous left ventricular assist devices, the association between preemptive MCS and procedural complications was concordant with the primary analysis and was accompanied by lower heterogeneity (OR 2.35; 95% CI 1.23–4.51; I²=6%). All-cause mortality and VT recurrence remained without significant differences (OR 0.80; 95% CI 0.40–1.62; I²=20% and OR 1.10; 95% CI 0.70–1.74; I²=28%, respectively), and the substantial heterogeneity observed for mortality in the primary analysis was attenuated. Procedural time remained longer among patients receiving MCS, although this estimate was derived from four studies and showed considerable heterogeneity (MD 63.5 min; 95% CI 3.6–123.4; I²=92%).

In leave-one-out sensitivity analyses, the association with procedural complications and the absence of differences in mortality and VT recurrence were consistent across all iterations. The substantial heterogeneity observed for mortality was attributable largely to the study of Turagam et al. [16], the omission of which reduced the I² statistic from 81% to 37%.^16^ The longer procedural time associated with preemptive MCS persisted throughout all iterations.

## Discussion

We conducted a meta-analysis of the available studies to evaluate the efficacy of preemptive MCS for VT ablation. We found that preemptive MCS was not associated with a significant difference in all-cause mortality or VT recurrence. However, it was accompanied by longer procedures and more frequent procedural complications. Although MCS may achieve periprocedural hemodynamic stability, this benefit may be offset by an increased risk of device access–site and procedural complications, which must be considered when evaluating the use of MCS during VT ablation.

Patients undergoing VT catheter ablation may experience procedural factors that contribute to hemodynamic decompensation such as catheter movements and pacing maneuvers, multiple electrical cardioversions, and fluid overload. These factors may compromise the hemodynamic stability needed for activation and entrainment mapping [3]. Of note, contemporary practice has shifted increasingly toward substrate-based mapping performed largely in sinus rhythm, with only brief VT induction during the procedure. Activation and entrainment mapping during sustained tachycardia is generally reserved for slower, hemodynamically tolerated VT. Although preemptive MCS was associated with longer procedural times in our analysis, conceivably reflecting more complete activation and entrainment mapping during sustained tachycardia, this was not accompanied by a lower rate of VT recurrence relative to ablation without MCS. This observation raises the possibility that the additional mapping facilitated by hemodynamic support does not necessarily confer superior arrhythmia control, particularly if ablation without MCS more often relied on substrate-based mapping performed in sinus rhythm. Because mapping strategy and ablation approach were not consistently reported across the included studies, this interpretation should be regarded as hypothesis-generating.

MCS devices have previously been used to achieve hemodynamic stability prior to ablation. A single-center study evaluated the use of ECMO for VT ablation compared with ablation without ECMO and found ECMO to be an appropriate tool to prevent acute procedural heart failure and reduce arrhythmic burden during follow-up.^11^ A prior systematic review found that patients receiving pLVAD support were generally higher-risk but demonstrated no significant differences in acute procedural success, VT recurrence, and mortality [17]. They also similarly found that pLVAD MCS support was associated with higher complications with longer procedural times [17]. An earlier meta-analysis of prophylactic mechanical support reported a comparable pattern: although preemptive support permitted induction of more ventricular tachycardias and a longer time mapping during tachycardia, these gains were not accompanied by differences in procedural success, VT recurrence, or short-term mortality [18]. Concordant findings were reported by a recent propensity score–matched analysis of 481 patients undergoing scar-related VT ablation, in which percutaneous left ventricular assist device support was associated with longer procedures and more frequent periprocedural complications—largely vascular complications and decompensated heart failure—without a corresponding improvement in long-term outcomes or VT recurrence [19]. An earlier single-center comparison observed similar 30-day mortality and arrhythmia-free survival between preemptively supported and unsupported patients, whereas rescue device insertion after hemodynamic collapse was associated with markedly higher mortality, consistent with the distinction between preemptive and rescue support that framed the present analysis [20]. Our study similarly demonstrates that procedural risks may be higher in patients receiving MCS, even though preemptive MCS is intended to support hemodynamically intolerant VT or anticipated hemodynamic compromise during ablation.

Our findings suggest that routine use of preemptive MCS for all patients undergoing VT ablation may not be warranted. Careful patient selection is therefore important when considering prophylactic hemodynamic support. MCS may remain valuable in high-risk patients in whom sustained VT mapping would otherwise be precluded by hemodynamic intolerance [4, 8]. In such cases, MCS may facilitate more complete mapping and improve long-term arrhythmia control, albeit with greater procedural complexity [5]. 

Risk stratification tools may help identify patients most likely to benefit from prophylactic MCS. The PAINESD score has been developed to predict the risk of acute hemodynamic collapse during VT ablation and incorporates clinical variables such as advanced age, ischemic cardiomyopathy, New York Heart Association functional class, ejection fraction, diabetes, and electrical storm [6]. Such tools may support individualized decisions regarding preemptive MCS rather than routine device placement. Prospective studies incorporating standardized risk stratification are needed to define the role of MCS in VT ablation. The MCS group carried a higher baseline predicted risk than the no-MCS group, with a mean PAINESD score of 15.7 compared with 10.5, yet all-cause mortality was not significantly higher. Although this pattern may reflect confounding by indication, with support reserved for sicker patients, the comparable mortality despite higher baseline risk cannot be interpreted as evidence of benefit, because the outcomes of these patients in the absence of MCS are unknown. This question merits evaluation in prospective, risk-stratified studies.

### Limitations

There are some limitations to our study. First, the available studies did not consistently report all complication subtypes, limiting our ability to perform detailed analyses of specific procedural complications. This limitation is inherent to retrospective studies, in which the relevant data are often incompletely reported. Second, in our delineation of various MCS devices, we opted to not include intra-aortic balloon pump (IABP) as a source of MCS, as IABP provides relatively limited hemodynamic support compared with other percutaneous MCS devices. Studies were excluded only when IABP was the sole modality of hemodynamic support; studies in which IABP was used alongside pLVAD or ECMO were retained and classified as mixed mechanical circulatory support [7]. This distinction is supported by multicenter data in which, relative to IABP, Impella or TandemHeart support enabled activation and entrainment mapping in a greater proportion of patients and required fewer rescue shocks, albeit without a difference in mortality or VT recurrence [21]. Additionally, many of the studies mixed IABP with other forms of MCS or in cohorts not using MCS, making it difficult to identify the impact of IABP for MCS. Lastly, significant heterogeneity among the included studies represents an important limitation. This heterogeneity may limit the applicability of the pooled effect, which may not represent individual study estimates. Potential sources of this heterogeneity include differences in the type of circulatory support device, baseline patient risk, ablation strategy, and the definitions applied to procedural and clinical outcomes. Consistent with a device-related contribution, restricting the analysis to studies using percutaneous left ventricular assist devices attenuated the heterogeneity observed for mortality, and leave-one-out analysis identified a single study as its principal driver. Importantly, although our eligibility criteria did not restrict study design, all included studies were observational and nonrandomized, and no randomized controlled trials were identified. The retrospective nature of the available evidence raises concern for selection bias and confounding by indication, as preemptive MCS was preferentially applied to higher-risk patients. These factors limit causal inference and may obscure a true treatment effect. The small number of studies, fewer than ten for each outcome, precluded formal assessment of publication bias using funnel-plot asymmetry or related statistical tests. In addition, a review protocol was not prospectively registered. Accordingly, these findings reflect the limitations of observational data and should not be interpreted as evidence against the use of preemptive MCS in appropriately selected high-risk patients; adequately powered randomized trials are needed to define its role.

## Conclusions

Our results suggest no clear overall benefit of routine preemptive MCS during VT ablation. Whether patients with specific comorbidities or structural abnormalities derive greater benefit from preemptive support than the broader VT ablation population merits further study. Larger, prospective studies with longer follow-up periods would help confirm these findings.

## Electronic Supplementary Material

Below is the link to the electronic supplementary material.

Supplementary Material 1 (DOCX 32.8 KB)

## Data Availability

Data Availability Statement: The datasets analyzed during the current study consist of aggregate data extracted from previously published articles. All extracted data supporting the findings of this study are included within the article and its supplementary materials. Source data are available from the original publications cited in the reference list.
