# Supplementary material for: Preemptive mechanical circulatory support in ventricular tachycardia ablation: A systematic review and meta-analysis
Source: J Interv Card Electrophysiol. 2026 Sep 22;69(6):1503–12. doi: 10.1007/s10840-026-02445-0 (PMC13645847; doi:10.1007/s10840-026-02445-0)
Supplement: Supplementary file 1 [file 10840_2026_2445_MOESM1_ESM.docx]

**Supplementary Table 1:** Per-study methodological characteristics of the included studies.

| **Study** | **MCS device and indication** | **Ablation strategy** | **Complications captured** | **MACE definition** | **Mortality ascertainment** |
| --- | --- | --- | --- | --- | --- |
| Aryana 2014^9^ | pLVAD (Impella 2.5); circulatory support for hemodynamically unstable VT | Activation and entrainment mapping during device-supported VT | Access-site injury; cardiac tamponade | Not natively defined; harmonized composite applied | Follow-up, mean 19 months |
| Bunch 2012^10^ | pVAD; alternative to substrate-based ablation to permit mapping during VT | MCS arm, mapping during induced VT; comparator arm, substrate-based ablation | Stroke; pericardial effusion; heart failure requiring ventilation | Not natively defined; harmonized composite applied | Follow-up, mean 9 months |
| Grimaldi 2021^11^ | VA-ECMO; hemodynamic support during ablation with instability | Mapping during ECMO support; post-ablation inducibility assessed | Femoral artery dissection or pseudoaneurysm; cannula dislodgement with hemorrhagic shock | Not natively defined; harmonized composite applied | Follow-up, 24 months |
| Kataoka 2023^12^ | Mixed MCS established for cardiogenic shock or refractory electrical storm and present before ablation; ablation performed under existing support | Urgent ablation; strategy per operator | Cardiac tamponade; anemia; hemolysis | Not natively defined; harmonized composite applied | Follow-up, approximately 3 months |
| Kusa 2017^13^ | pLVAD; VT ablation with pLVAD support | Mapping during device-supported VT | Pericardial effusion; vascular complications; heart failure exacerbation | Not natively defined; harmonized composite applied | Follow-up, 7 months |
| Muser 2018^14^ | pLVAD; prophylactic use in high-risk patients (propensity-matched) | Mapping during device-supported VT | Pericardial tamponade or effusion; femoral artery occlusion; device fracture on removal; embolic stroke; deep vein thrombosis | Not natively defined; harmonized composite applied | Follow-up, 9 months |
| Sroubek 2023^15^ | Impella 5.0/5.5; high-risk advanced heart failure (first-in-human) | Mapping during device-supported VT | Access-site hematoma; infected deep vein thrombosis; pulseless electrical activity arrest during placement; pericardial tamponade; refractory shock | Not natively defined; harmonized composite applied | Follow-up, approximately 15 months |
| Turagam 2017^16^ | Mixed MCS across centers (multicenter collaborative) | Variable across centers; mapping during supported VT | Reported in aggregate only (not itemized) | Not natively defined; harmonized composite applied | Follow-up, 17.3 months |

One study (Turagam et al) reported complications only in aggregate. A 30-day major adverse cardiovascular event (MACE) composite was not uniformly defined by the included studies; a harmonized composite of death, myocardial infarction, stroke, and revascularization was applied during data extraction. All-cause mortality was ascertained at each study’s reported follow-up interval rather than at a uniform time point. pLVAD = percutaneous left ventricular assist device; pVAD = percutaneous ventricular assist device; VA-ECMO = venoarterial extracorporeal membrane oxygenation; VT = ventricular tachycardia. **Supplementary Table 2:** Database-specific search strategies.

| **Database (platform)** | **Date searched** | **Search string** | **Limits or filters** | **Records retrieved** |
| --- | --- | --- | --- | --- |
| PubMed (U.S. National Library of Medicine) | Database inception through May 2026 | ("Ablation") AND ("mechanical circulatory support" OR "ecmo" OR "impella") NOT (Review[publication type]) | Review articles excluded | 119 |
| Web of Science (Clarivate) | Database inception through May 2026 | (ablation) AND ("mechanical circulatory support" OR "ecmo" OR "impella") | None | 163 |
| Embase (Elsevier) | Database inception through May 2026 | ('ablation'/exp OR 'ablation') AND (('mechanical circulatory support'/exp OR 'mechanical circulatory support') OR ('extracorporeal membrane oxygenation'/exp OR 'extracorporeal membrane oxygenation' OR ecmo) OR ('impella'/exp OR impella)) | None | 61 |
